# Engineering xanthophyll esterification: LbXES2 mediates zeaxanthin dipalmitate biosynthesis and remodels fruit flavor metabolism

**DOI:** 10.1093/hr/uhag228

**Published:** 2026-06-10

**Authors:** Hong Zhang, Huanjun Li, Dexin Lyu, Jin Shi, Ren Chen, Shan Lu, Fulei Mo, Guilian Mao, Aoxue Wang, Wanqi Liang

**Affiliations:** School of Life Sciences and Biotechnology, Shanghai Jiao Tong University, Shanghai 200240, China; School of Life Science, Ningxia University, Yinchuan, Ningxia 750021, China; School of Life Sciences and Biotechnology, Shanghai Jiao Tong University, Shanghai 200240, China; State Key Laboratory of Pharmaceutical Biotechnology, School of Life Sciences, Nanjing University, Nanjing 210023, China; School of Life Sciences and Biotechnology, Shanghai Jiao Tong University, Shanghai 200240, China; School of Life Science, Ningxia University, Yinchuan, Ningxia 750021, China; State Key Laboratory of Pharmaceutical Biotechnology, School of Life Sciences, Nanjing University, Nanjing 210023, China; College of Horticulture and Landscape Architecture, Northeast Agricultural University, Harbin 150030, China; School of Life Science, Ningxia University, Yinchuan, Ningxia 750021, China; College of Horticulture and Landscape Architecture, Northeast Agricultural University, Harbin 150030, China; School of Life Sciences and Biotechnology, Shanghai Jiao Tong University, Shanghai 200240, China; Yazhou Bay Institute of Deepsea Sci-Tech, Shanghai Jiao Tong University, Sanya 572024, China

## Abstract

*Lycium barbarum* fruits are valued for bioactive carotenoids, yet the enzymatic basis underlying accumulation of their hallmark xanthophyll diester, zeaxanthin dipalmitate (ZD), remains unclear. Biosynthesis of ZD requires two sequential reactions: β-carotene hydroxylation catalyzed by β-carotene hydroxylase (BCH) to produce zeaxanthin, followed by xanthophyll esterification mediated by xanthophyll esterase (XES). However, the specific genes responsible for ZD formation in *L. barbarum* have not been functionally defined. Here, we integrated genome mining, phylogenetics, developmental metabolite profiling, tissue- and stage-resolved expression analysis, and functional validation using *Escherichia coli* complementation, transient expression in *L. barbarum* and tobacco, and fruit-specific co-expression in tomato. Carotenoid profiling revealed a ripening-associated transition from free xanthophylls to esters. Among the candidates, *LbBCH1* and *LbXES2* showed fruit-preferential expression, positively correlated with ZD accumulation, and both encoded plastid-localized proteins. In *E. coli* complementation assays, LbBCH1 converted β-carotene to zeaxanthin, and LbXES2 further catalyzed the formation of ZD from zeaxanthin. Transient expression assays showed that LbXES2 could esterify multiple xanthophylls and exhibited a preference for palmitoylation, while ZD production was enhanced by LbBCH1-mediated zeaxanthin supply. In tomato, fruit-specific co-expression of *LbBCH1* and *LbXES2* enabled *de novo* biosynthesis of xanthophyll ester, elevated total carotenoids, promoted chromoplast and plastoglobule development, and altered sugars, organic acids, and carotenoid-derived volatiles. Collectively, these results identify LbXES2 as a xanthophyll esterification enzyme associated with ZD biosynthesis and support a functional role for LbBCH1-mediated zeaxanthin supply in this process. Our study provides molecular tools and a heterologous engineering strategy for producing high-value esterified carotenoids in fleshy fruits.

## Introduction

*Lycium barbarum* L., a deciduous thorny shrub of the Solanaceae family, produces nutrient-dense fruits commonly known as goji berries (wolfberries). *L. barbarum* is widely appreciated for its high levels of bioactive phytochemicals, including carotenoids, flavonoids, and phenolic acids, which collectively confer strong antioxidant and anti-inflammatory activities [1, 2]. These compounds are implicated in alleviating oxidative stress and chronic inflammation, two key pathological drivers of metabolic disorders such as diabetes, obesity, and cardiovascular disease [3]. During fruit maturation, carotenoid accumulation increases dynamically and is closely associated with the characteristic red pigmentation of ripe goji berries.

Carotenoids are synthesized from the universal C5 precursors isopentenyl diphosphate and dimethylallyl diphosphate, which are condensed to form geranylgeranyl diphosphate (GGPP). In plastids, GGPP is converted to all-*trans*-lycopene through sequential reactions catalyzed by phytoene synthase, phytoene desaturase, ζ-carotene isomerase, ζ-carotene desaturase, and carotene isomerase. Lycopene is then cyclized to generate either β-carotene or α-carotene [4]. β-Carotene is subsequently hydroxylated by β-carotene hydroxylase (BCH) to produce zeaxanthin [5]. Further epoxidation and structural modification of zeaxanthin yield xanthophylls such as antheraxanthin, violaxanthin, and neoxanthin [6]. In nonphotosynthetic tissues, zeaxanthin and related xanthophylls are frequently present as free forms, monoesters, or diesters, in which fatty acids are esterified to one or both hydroxyl groups of the carotenoid backbone.

A distinctive feature of *L. barbarum* fruit is the predominant accumulation of zeaxanthin as the diester zeaxanthin dipalmitate (ZD), which typically constitutes approximately one-third to one-half of the total carotenoid pool and represents a distinctive feature that is rarely observed in most other species [7, 8]. ZD has attracted considerable interest due to reported antioxidant and anti-inflammatory activities and associated protective effects in disorders related to oxidative stress. In particular, ZD has been reported to exert hepatoprotective effects in nonalcoholic fatty liver disease, alcoholic liver disease, liver cirrhosis, and liver failure [9–12]. In the visual system, wolfberry-derived ZD can delay retinal degeneration in a retinitis pigmentosa model through modulation of signal transducer and activator of transcription 3, chemokine (C-C motif) ligand 2 (CCL2), and mitogen-activated protein kinase signaling [13], and dietary supplementation with ZD improves photoreceptor survival, retinal responses, and visual acuity in degenerative mice [14].

Xanthophyll esterification is generally catalyzed by xanthophyll esterase (XES) or related xanthophyll acyltransferases (XATs), which transfer fatty acyl groups to hydroxyl moieties on xanthophyll substrates [15]. Several functionally validated XES enzymes—such as PYP1, IoXES, TeXES, BrWF3/BrPYP, BjA02.PC1, and BjB04.PC2—share homology with the Arabidopsis esterase/lipase/thioesterase family and have been linked to carotenoid esterification in flowers or fruits of tomato (*Solanum lycopersicum*), morning glory (*Ipomoea obscura*), marigold (*Tagetes* spp.), carnation (*Dianthus caryophyllus*), Chinese cabbage (*Brassica rapa*), and mustard (*Brassica juncea*) [16–22]. Despite the abundance and high nutritional value of ZD in *L. barbarum*, the enzymes responsible for ZD biosynthesis in this species remain largely unresolved. In particular, it is still unclear which xanthophyll esterification enzyme mediates ZD formation in *L. barbarum* fruit and to what extent ZD accumulation depends on xanthophyll esterification capacity versus zeaxanthin precursor supply (Fig. S1). A candidate gene (*Lba07g02085*), encoding a ttLC_FACS_AEE21-like domain protein, has been proposed from in silico analyzes [23], but functional validation, a coherent enzymatic framework, and direct experimental evidence for the complete ZD biosynthetic pathway in *L. barbarum* are still lacking.

Given these unresolved questions, together with the technical challenges of stable genetic analysis in *L. barbarum*, due to limited transformation efficiency and a long juvenile period before fruiting, heterologous validation systems provide a particularly valuable approach. Therefore, this study aimed to identify and functionally characterize the xanthophyll esterase involved in ZD biosynthesis in *L. barbarum*, assess the contribution of LbBCH1-mediated zeaxanthin supply to ZD accumulation, determine the substrate preference of the characterized LbXES2, and reconstruct the ZD biosynthetic pathway in tomato to evaluate its transferability and impacts on carotenoid accumulation and flavor-related metabolism.

## Results

### Dynamic accumulation of xanthophyll esters during *L. barbarum* fruit ripening

To systematically analyze carotenoid dynamics, we categorized the ripening process of red wolfberry fruits into four developmental stages (S1–S4) and quantified their carotenoid composition at each stage. Carotenoid profiles displayed pronounced tissue- and stage-dependent patterns in both leaves and fruits (Fig. 1a and b). In leaves, lutein—an isomer of zeaxanthin that shares an identical molecular formula yet differs in ring structures and hydroxyl configurations—accumulated to levels significantly higher than those of other xanthophylls (*P* < 0.01), reaching 13-, 22-, and 368-fold of violaxanthin, antheraxanthin, and zeaxanthin, respectively (Fig. 1a; Fig. S2a). In contrast, zeaxanthin (the direct precursor of ZD) was present only at very low abundance in leaves.

**Figure 1:**
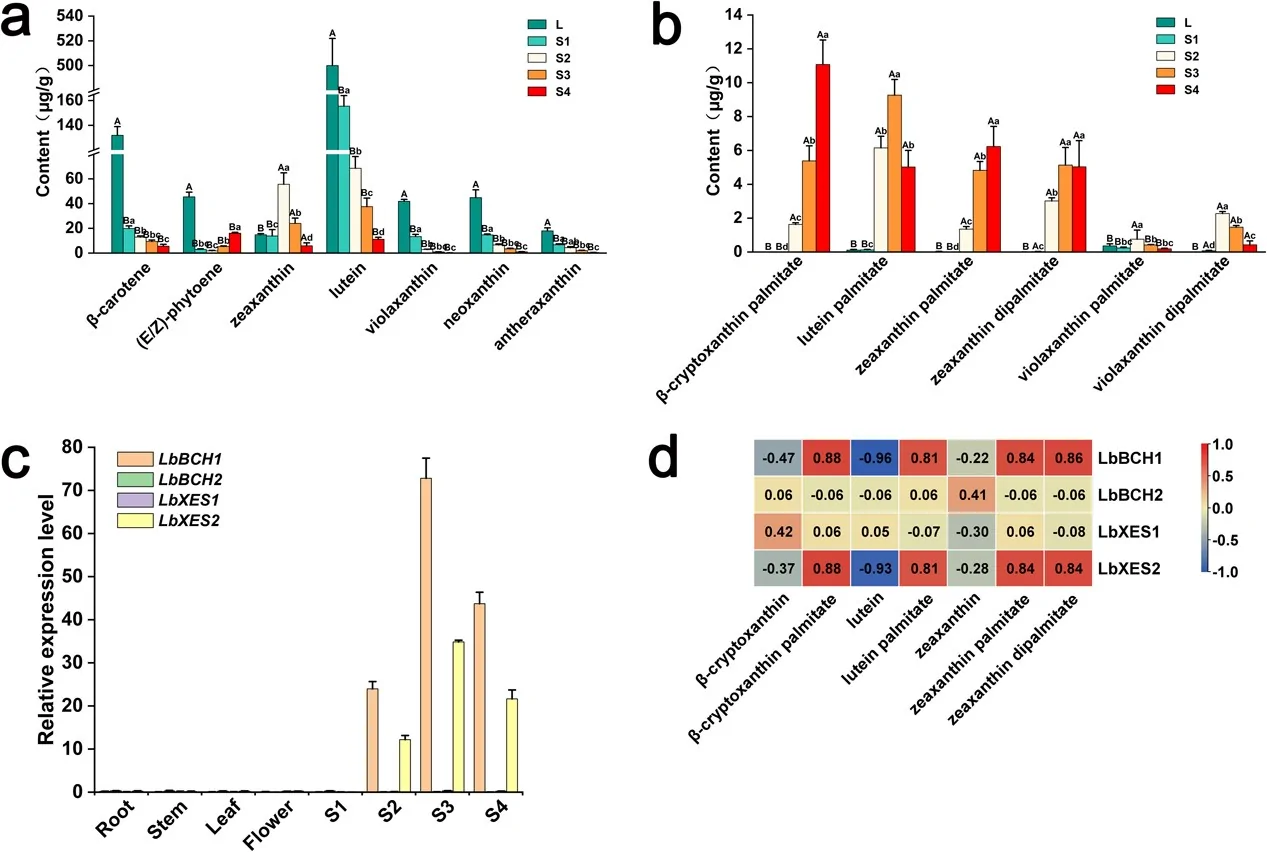
Dynamics of carotenoid accumulation and *LbBCH/LbXES* expression in *L. barbarum* leaves and fruits across developmental stages. (a) Contents of free carotenoids in leaves and at different fruit developmental stages. (b) Contents of esterified carotenoids in leaves and at different fruit developmental stages. Values are presented as mean ± SE (*n* = 3). Statistical significance was evaluated by two-way ANOVA followed by Tukey’s multiple comparison test. Different capital letters represent significant differences in carotenoid contents between leaves and fruits (*P* < 0.05), while lowercase letters represent significant differences in carotenoid contents among fruits at different developmental stages (*P* < 0.05). (c) Expression profiles of *LbXESs* and *LbBCHs*, key genes potentially involved in carotenoid biosynthesis in leaves and fruits at different developmental stages. Error bars represent mean ± SD (*n* = 3). (d) Heatmap of normalized transcript abundance (transcripts per million) and metabolite–gene expression correlations. Pearson correlation coefficients are shown.

During fruit development, zeaxanthin increased from stage S1, peaked at the turning stage (S2, 55.70 μg/g), and subsequently declined (Fig. 1a). In contrast, lutein was highest at S1 and decreased to a level comparable to that of zeaxanthin thereafter (Fig. S2b). Zeaxanthin dipalmitate and lutein palmitate were first detected at S2. Zeaxanthin dipalmitate levels increased progressively during fruit ripening, reaching a maximum (5.02 μg/g, dry weight) at the fully ripe stage, while lutein palmitate remained at a high level in later stages. Importantly, neither esterified form was detected in leaves or in stage S1 fruits (Fig. 1b). Collectively, these data indicate a clear developmental transition from free xanthophylls to esterified derivatives during fruit ripening.

### Phylogenetic analysis characterizes LbBCH and LbXES as putative enzymes for zeaxanthin dipalmitate formation

Homologous sequences of LbXES were first retrieved from the *L. barbarum* genome database via BLAST searches using *Arabidopsis thaliana* At1g54570 and *Solanum lycopersicum* SlPYP1 (Solyc01g098110.2) as queries, and LbBCH homologous sequences were further identified by means of functional annotation against this database. Conversion of β-carotene to ZD requires β-carotene hydroxylation and subsequent xanthophyll acylation, catalyzed primarily by BCH and XES, respectively. Phylogenetic analysis of BCH across representative plant species identified two BCH homologs in *L. barbarum*, designated LbBCH1 and LbBCH2, which segregated into distinct clades (Fig. S3a). LbBCH1 clustered with tomato SlBCH1, whereas LbBCH2 grouped with SlBCH2, and both clades showed high similarity to AcBCH from kiwifruit (Fig. S4). Functional relevance of this lineage is supported by a study in kiwifruit, where silencing of *AcBCH1* reduces zeaxanthin accumulation while increasing β-carotene content [24].

The carotenoid biosynthetic pathway in higher plants has been well established [4], and xanthophyll ester formation occurs downstream of xanthophyll biosynthesis (Fig. S1). Several acyltransferases, including tomato PYP1, pepper rsAcT, *Brassica juncea* BjPCs*,* and *Ipomoea obscura* XES, have been shown to catalyze xanthophyll ester formation. Phylogenetic analysis placed LbXES1 and LbXES2 in close proximity to these characterized carotenoid acyltransferases (Fig. S3b). Consistent with this, motif analysis indicated that LbXES proteins contain the canonical signatures of benzyl alcohol O-acetyltransferase, anthocyanin O-hydroxycinnamoyltransferase, anthranilate N-hydroxycinnamoyl/benzoyltransferase, deacetylvindoline 4-O-acetyltransferase (BAHD) acyltransferases, alongside a domain homologous to diacylglycerol acyltransferases (DGAT) (Fig. S5).

### Fruit-specific expression and chloroplast localization identify LbBCH1 and LbXES2 as key players in xanthophyll ester formation

To identify candidate members involved in ZD biosynthesis in fruits, we examined transcript abundances of *LbBCHs* and *LbXESs* by quantitative real-time PCR (qRT-PCR) in multiple tissues of *L. barbarum*, including roots, stems, leaves, flowers, and fruits. *LbBCH1* and *LbXES2* were predominantly expressed in fruits, with only trace transcript levels detected in other organs. In contrast, *LbBCH2* and *LbXES1* exhibited low but ubiquitous expression across all tissues examined (Fig. 1c).

To evaluate the relationship between gene expression and metabolite accumulation, transcript levels were further measured in the same samples used for metabolite profiling. Both *LbBCH1* and *LbXES2* were markedly upregulated in S2–S4 fruits compared with stage S1 fruits and leaves, with maximal expression at S3 (Fig. 1c). Correlation analysis revealed significant positive associations between transcript levels and carotenoid accumulation, particularly between *LbBCH1* and *LbXES2* expression and ZD content (Fig. 1d), suggesting that *LbBCH1* and *LbXES2* play a role in fruit carotenoid biosynthesis and esterification.

In plants, carotenoid biosynthesis occurs exclusively in plastids, where the methylerythritol 4-phosphate pathway directs carbon flux toward isoprenoid precursor synthesis. Bioinformatic analysis using TargetP predicted plastid localization for both LbBCH1/2 and LbXES1/2. We generated C-terminal green fluorescent protein (GFP) fusions for each candidate and transiently expressed the constructs in *N. benthamiana* leaves for subcellular localization analysis. Fluorescence microscopy observations showed that LbBCH1 and both LbXES1/2 localized to chloroplasts (Fig. 2), which is in accordance with the compartmentation of xanthophylls and fatty acids within plastids. Together, the fruit-specific expression patterns of *LbBCH1* and *LbXES2*, combined with the plastid localization of their cognate proteins, support their proposed biochemical function in the biosynthesis and esterification of carotenoids during fruit ripening in *L. barbarum*.

**Figure 2:**
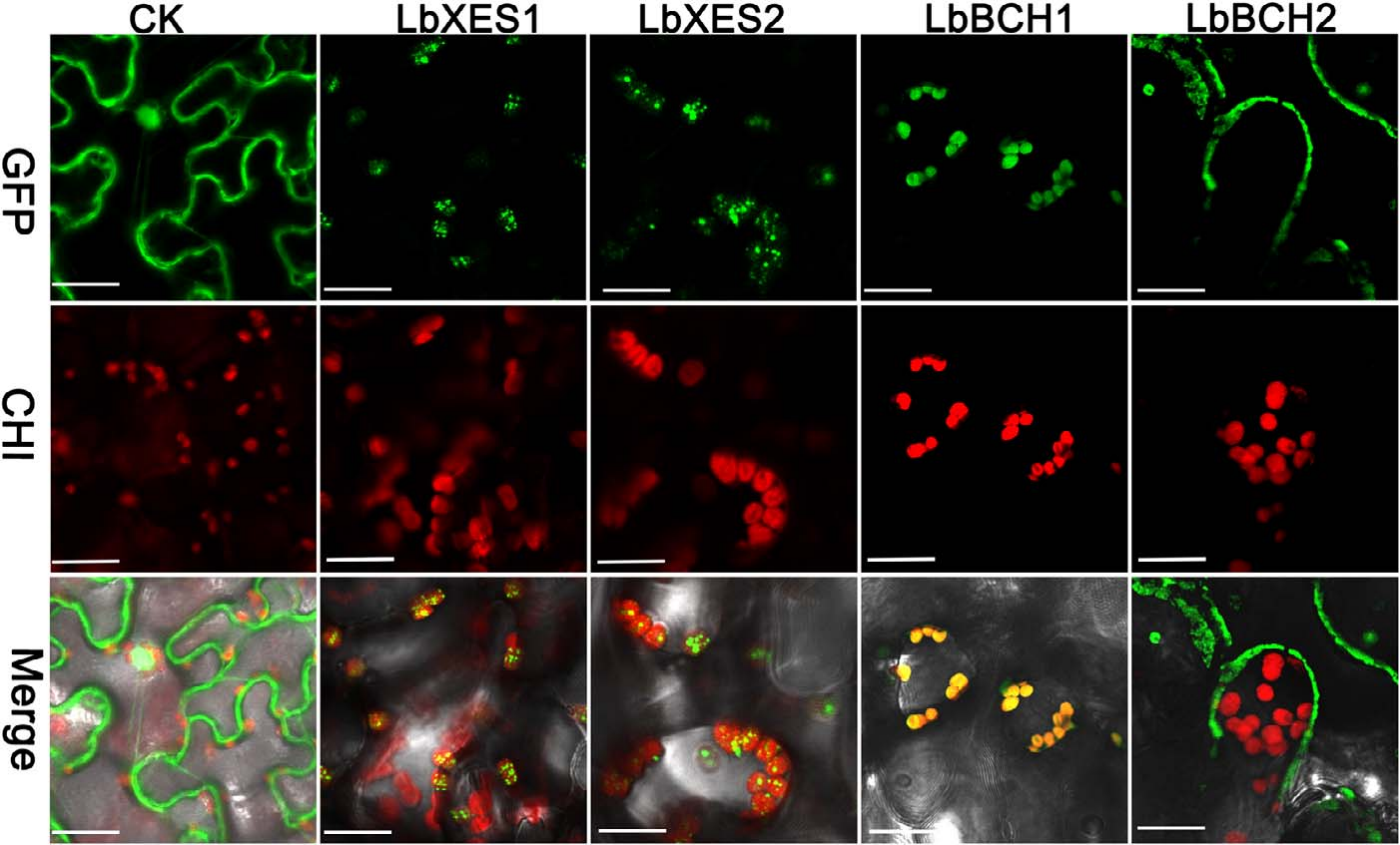
Subcellular localization of LbBCHs and LbXESs in *N. benthamiana* leaves. Representative confocal images show plastid localization of the GFP-tagged proteins. Top, GFP fluorescence. Middle, chlorophyll autofluorescence. Bottom, merged GFP and chlorophyll signals overlaid on the bright-field image. Scale Bar = 20 μm.

### Functional complementation of LbBCH1 and LbXES2 in *E. coli* defines their roles in zeaxanthin dipalmitate biosynthesis

To verify the biochemical activities of LbBCH1 and LbXES2 in the carotenoid biosynthetic pathway, their respective coding sequences (CDS) without signal peptides were cloned into the pET-32b vector, and an *E. coli*-based pigment complementation assay was performed [25, 26]. The resulting recombinant plasmid pET-LbBCH1 was co-transformed into *E. coli* BL21(DE3) with pAC-BETA, a plasmid harboring the carotenoid biosynthetic genes *crtE*, *crtB*, *crtI*, and *crtY* from Pantoea agglomerans, which enables β-carotene production in *E. coli*. For LbXES2, pET-LbXES2 was co-transformed into *E. coli* BL21(DE3) with pAC-ZEAX, which contains genes *crtE*, *crtB*, *crtI*, *crtY*, and *crtZ* and thus enables zeaxanthin accumulation in *E. coli*. Following induction, bacterial cells were harvested, lysed with acetone, and analyzed by liquid chromatography–mass spectrometry (LC–MS). A distinct zeaxanthin peak was consistently detected in cells expressing LbBCH1, but was absent in negative controls (Fig. 3a), confirming that LbBCH1 catalyzes the conversion of β-carotene to zeaxanthin. Similarly, a unique ZD peak was detected exclusively in cells expressing LbXES2 (Fig. 3b), demonstrating that LbXES2 mediates the acylation of zeaxanthin to form ZD.

**Figure 3:**
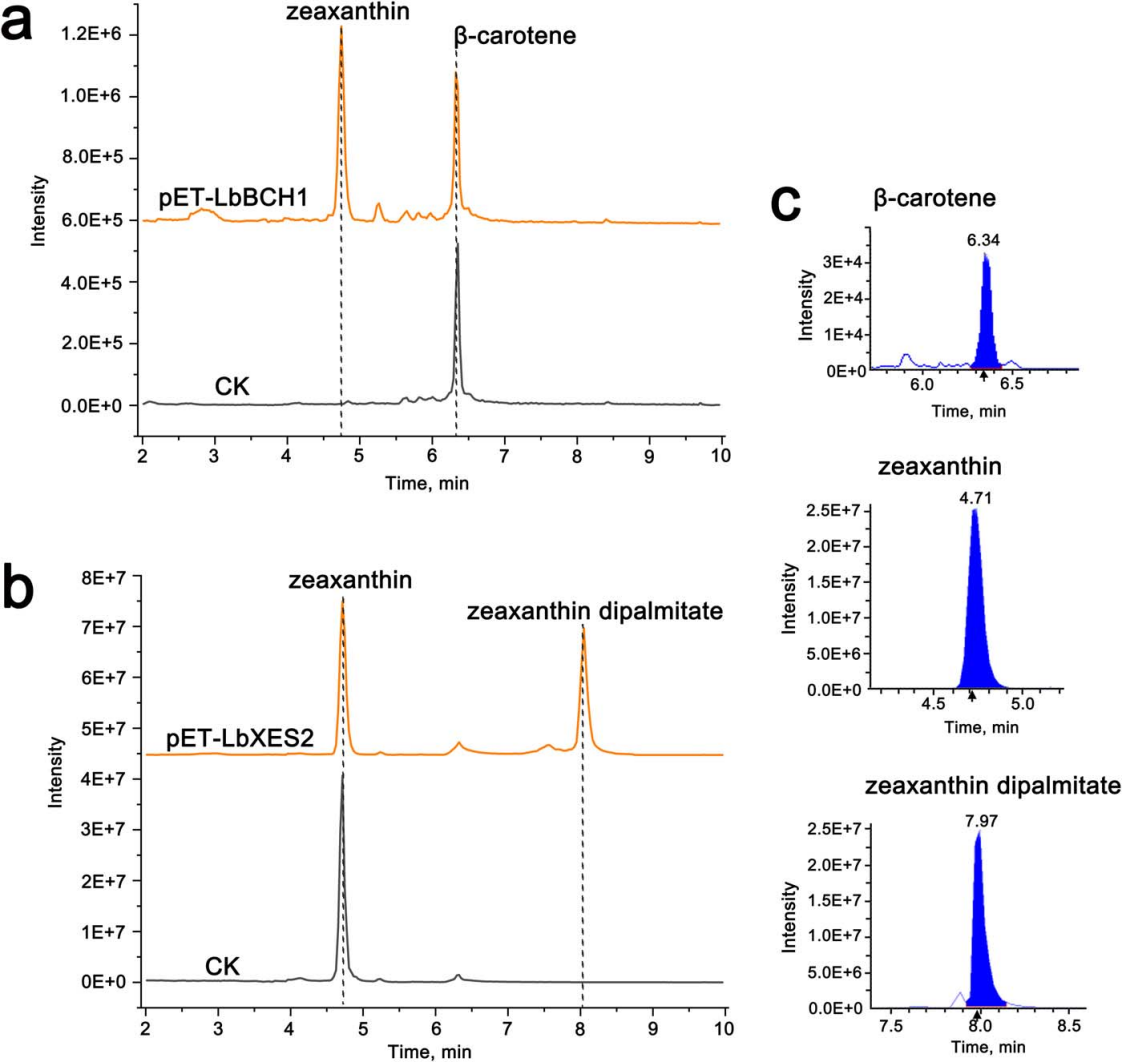
LC–MS analysis of carotenoids in *E. coli* expressing *LbBCH1* and *LbXES2*. (a) Chromatograms of *E. coli* expressing pET-LbBCH1 and the empty vector control (CK). The peaks corresponding to zeaxanthin and β-carotene are marked. ‘CK’ represents *E. coli* transformed with pAC-BETA; ‘pET-LbBCH1’ represents *E. coli* co-transformed with pAC-BETA and pET-LbBCH1. (b) Chromatograms of *E. coli* expressing pET-LbXES2 and the empty vector control (CK). The peaks corresponding to zeaxanthin and ZD are marked. (c) The integral correction diagram of β-carotene, zeaxanthin, and ZD in *E. coli*.

### Enhanced zeaxanthin supply by LbBCH1 boosts LbXES2-mediated zeaxanthin dipalmitate formation

Given the high abundance of free xanthophylls and negligible accumulation of their esterified forms in leaves, we employed a transient expression system using the high-efficiency pEAQ plant expression vector via an agroinfiltration method to characterize the biochemical function of LbXES2 in *L. barbarum* leaves [27, 28]. Expression of *LbXES2* induced substantial metabolic changes in infiltrated leaves (Fig. 4a and b). We observed a significant 24-fold increase in lutein palmitate accumulation compared with the empty-vector control (Fig. 4a and b). Lutein palmitate and zeaxanthin palmitate are monohydroxy carotenoid esters formed by esterification of the C3/C3′ hydroxyl groups with palmitic acid (C16:0). Notably, zeaxanthin palmitate, which was undetectable in control leaves, became clearly detectable following *LbXES2* expression. In addition, LbXES2 promoted accumulation of a broad spectrum of xanthophyll esters, including violaxanthin dipalmitate (Vio-diP), antheraxanthin dipalmitate, lutein dimyristate, and lutein dipalmitate (Fig. 4b). These results demonstrate that *LbXES2* encodes a functional acyltransferase capable of converting free xanthophylls to their esterified derivatives in planta.

**Figure 4:**
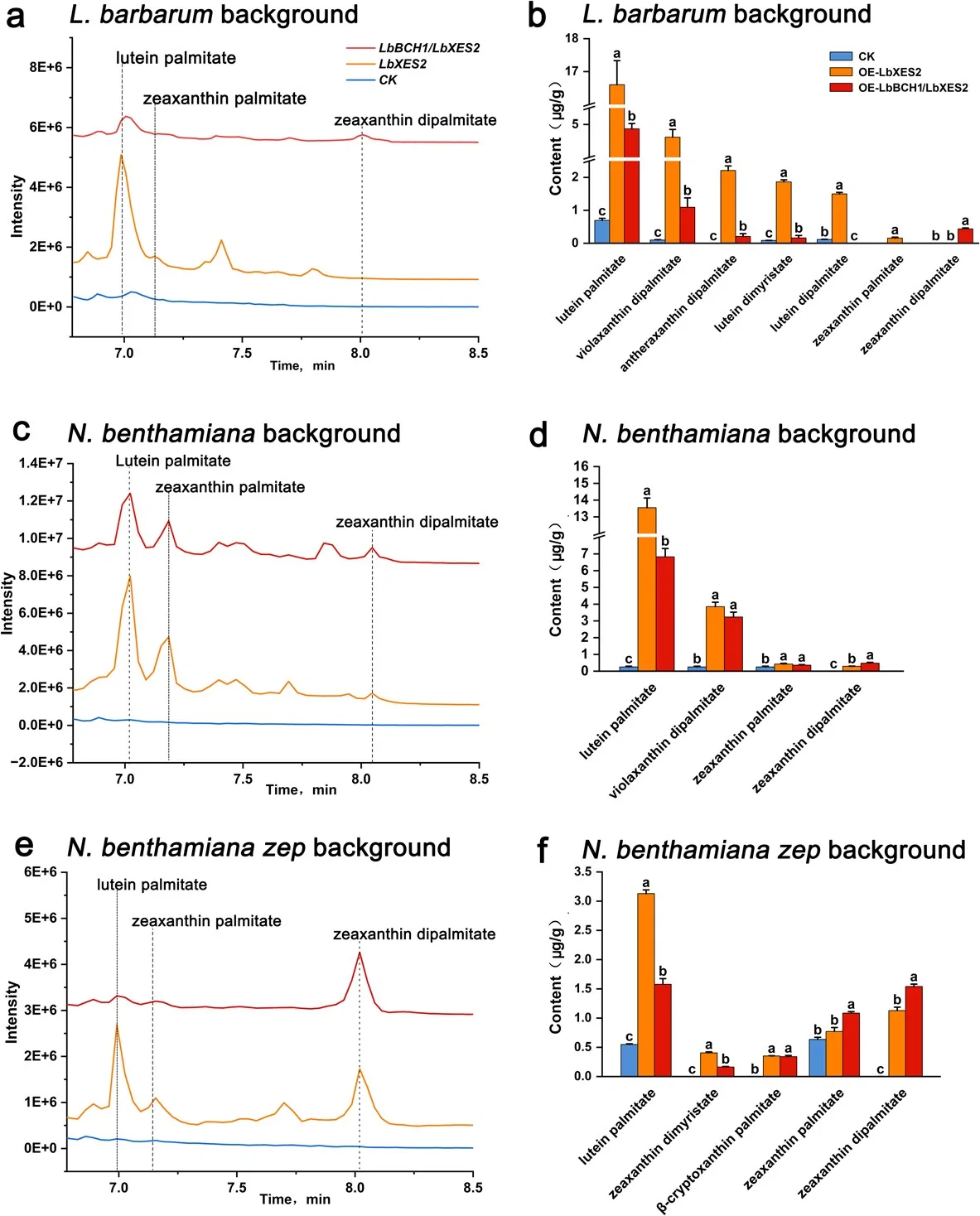
LbXES2 esterifies multiple xanthophyll substrates, and ZD production is substrate dependent. (a, c, e): LC–MS chromatograms of carotenoids from *L. barbarum* leaves (a), *N. benthamiana* leaves (c), and *N. benthamiana zep* mutant leaves (e) transiently expressing the empty vector control (CK), LbXES2, or co-expressing LbXES2 and LbBCH1. (b, d, f): Quantification of carotenoid esters (μg/g DW) corresponding to panels (a), (c), and (e), respectively. Data are presented as mean ± SE (*n* = 3 biological replicates). Statistical significance was assessed by one-way ANOVA followed by Tukey’s multiple comparison test. Different letters above the error bars indicate significant differences at *P* < 0.05.

Although zeaxanthin palmitate accumulated upon *LbXES2* expression, ZD was not detected in *L. barbarum* leaves, likely due to limited zeaxanthin availability compared with other xanthophyll substrates. To increase zeaxanthin supply, *LbBCH1* was co-expressed with *LbXES2*, resulting in a marked accumulation of ZD (0.5 μg/g; Fig. 4a and b; Supplementary Table S1). Parallel transient expression assays in wild-type *N. benthamiana* leaves further demonstrated robust diesterification activity of LbXES2, with co-expression of LbBCH1 enhancing ZD production (Fig. 4c and d). To further substantiate LbXES2 function, we performed transient assays in an *N. benthamiana zep* mutant, which is deficient in antheraxanthin and violaxanthin biosynthesis downstream of zeaxanthin due to the absence of zeaxanthin epoxidase (ZEP). Metabolic profiling revealed consistent accumulation of ZD, zeaxanthin dimyristate, and β-cryptoxanthin palmitate in both *LbXES2*-expression and *LbXES2/LbBCH1* co-expression lines, whereas these compounds were undetectable in control leaves. In particular, ZD reached 1.13 μg/g in *LbXES2* overexpression (OE) lines and 1.54 μg/g in *LbXES2/LbBCH1* co-expression lines (Fig. 4e and f; Supplementary Table S3). Together, these data support an evolutionarily conserved role for LbXES2 in carotenoid esterification and demonstrate that substrate supply (via LbBCH1) can be rate-limiting for zeaxanthin diester accumulation.

### Co-expression of LbBCH1 and LbXES2 enhances carotenoid accumulation and alters chromoplast ultrastructure in transgenic fruits

To engineer esterified carotenoid biosynthesis in tomato fruits, we generated transgenic lines co-expressing *LbXES2* and *LbBCH1* under the control of the fruit-specific E8 promoter (Fig. S6). Transformation was performed in *Solanum lycopersicum* var. *cerasiforme* ‘Yellow Fruit Cherry Tomato’, a cultivar that naturally accumulates negligible levels of xanthophyll esters and therefore provides a suitable background to evaluate heterologous esterification capacity (Supplementary Table S4). Multiple independent transgenic lines were obtained, and two lines showing stable and reproducible phenotypes were selected for detailed analysis. Compared with the wild type, ripe fruits of both transgenic lines displayed a clear color change from pale orange to orange-red (Fig. 5a).

**Figure 5:**
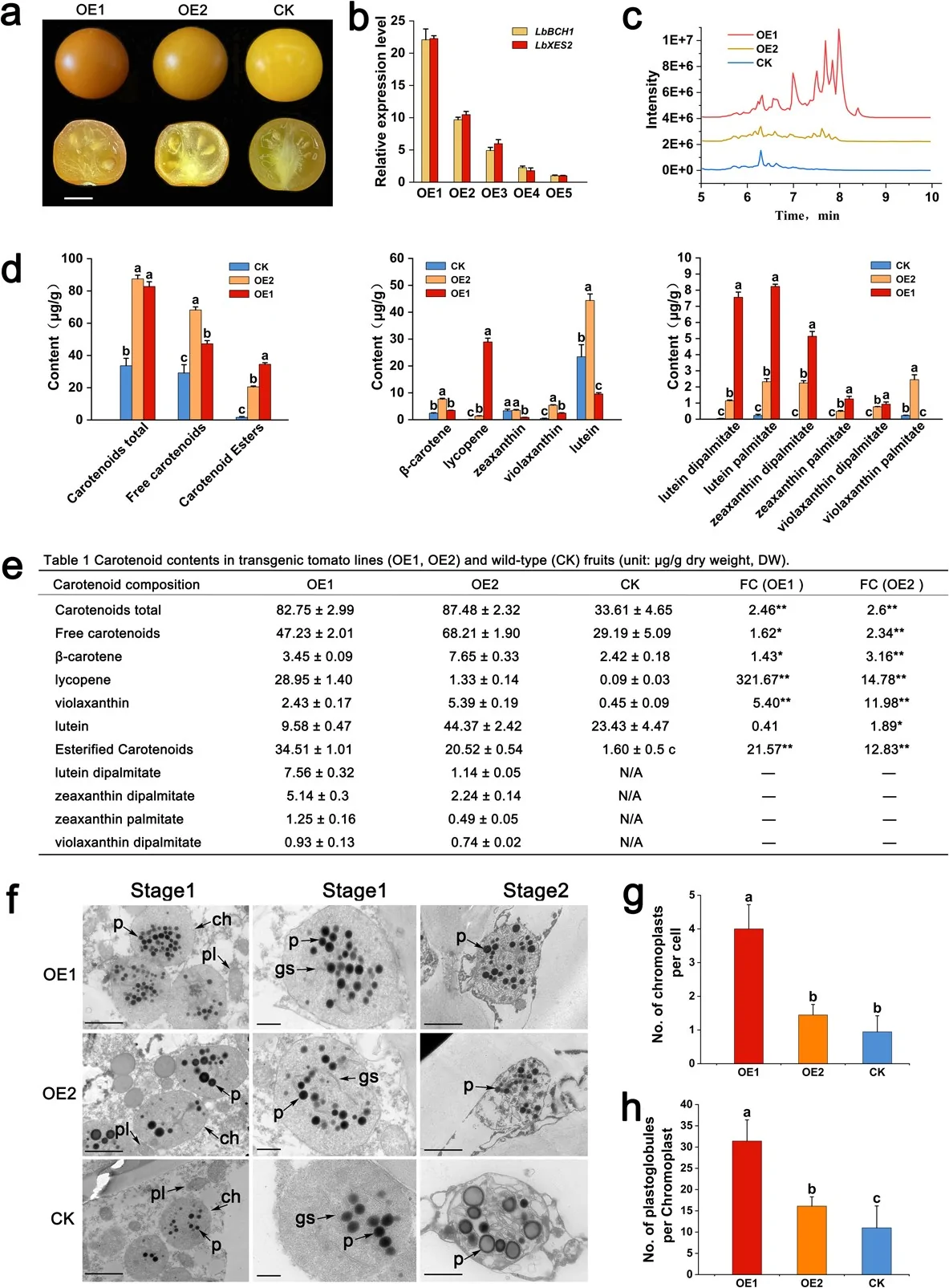
Phenotypic appearance and carotenoid profiles of transgenic tomato fruits co-expressing *LbBCH1* and *LbXES2*. (a) Fruit morphology of the control (CK, ‘Yellow Fruit Cherry Tomato’ wild type) and *LbBCH1/LbXES2* co-expressing lines (OE1 and OE2) under the control of the E8 promoter. Scale bar, 1 cm. (b) Relative expression levels of *LbBCH1* and *LbXES2* in transgenic lines. Because no tomato *LbBCH1* and *LbXES2* transcript was present in non-transgenic plants, the expression of *LbBCH1* and *LbXES2* in OE line OE5 was set to 1. Values are mean ± SD of three biological replicates. (c) LC–MS chromatograms of carotenoids from control (CK) and transgenic (OE1 and OE2) fruits. (d) Quantification of carotenoids in control (CK) and transgenic lines (OE1 and OE2). Different letters above the error bars indicate significant differences at *P* <0.05. (e) Accumulation of total, free and esterified carotenoids in wild-type (CK) and transgenic (OE1, OE2) tomato fruits. Data are means ± SD. The embedded Table 1 shows exact contents and fold changes relative to CK. Compounds not detected in CK (N/A) were set to 0 for statistical analysis. Statistical significance was determined by one-way ANOVA followed by Tukey’s test: ^*^P <0.05, ^**^P <0.01, ns: not significant. (f) Representative transmission electron microscopy images from OE1 and CK fruit. Ch, chromoplast; pl, plastid; P, plastoglobules; gs, granal stacks; Bars = 1 μm. (g) The number of chromoplasts per cell in each genotype was counted (*n* = 15 fruit cells). (h) Average number of plastoglobules per plastid in each genotype was counted (*n* = 15 plastids). Fruits of T₁ transgenic and non-transgenic control (CK) plants were collected at two advanced ripening stages: breaker + 20 d (stage 1) and breaker + 25 d (stage 2).

Based on RT-qPCR analysis confirming robust OE of both *LbXES2* and *LbBCH1* in transgenic tomato fruits relative to wild-type (WT) controls (Fig. 5b), lines OE1 and OE2 were selected for detailed carotenoid profiling. Targeted profiling revealed that fruits from *LbXES2*/*LbBCH1* transgenic lines accumulated multiple carotenoid esters, including zeaxanthin palmitate, ZD, lutein dipalmitate, and violaxanthin dipalmitate, none of which were detectable in WT fruits (Fig. 5c and d). Total carotenoids ester levels reached up to 34.51 μg/g dry weight (DW), corresponding to an approximately 22-fold increase over WT (Fig. 5d and e). Notably, nutritionally relevant zeaxanthin palmitate and ZD accumulated to 2.24 and 5.14 μg/g DW, respectively, whereas both compounds were below the detection limit in WT fruits (Fig. 5d and e).

In addition to the emergence of esterified carotenoids, transgenic fruits exhibited a significant increase in total free carotenoids, reaching 68.21 μg/g DW, which represents a 2.3-fold increase compared with WT. Individual free carotenoids displayed differential accumulation, with significant increases in β-carotene (~3.2-fold), lycopene (~321-fold), violaxanthin (~11.9-fold), and lutein (~1.9-fold) in transgenic fruits (Fig. 5d and e). Consequently, total carotenoids increased 2.6-fold to 87.48 μg/g DW in transgenic fruits relative to WT (Fig. 5d and e). Collectively, these results demonstrate that co-expression of *LbBCH1* and *LbXES2* enables efficient heterologous production and accumulation of xanthophyll esters in tomato fruits, establishing a robust platform for metabolic engineering of high-value esterified carotenoids in heterologous crop species.

Given that carotenoid biosynthesis and storage rely on chromoplast development, transmission electron microscopy (TEM) was employed to characterize chromoplast ultrastructure and plastoglobule biogenesis in transgenic tomato fruits. Both OE1 and OE2 lines displayed significantly increased numbers of chromoplasts per cell and plastoglobules per chromoplast, with higher electron density compared to the wild type (CK). Notably, at Stage 2, chromoplasts in CK fruits contained large, vacuolated plastoglobules, whereas those in transgenic lines maintained compact, nonvacuolated, and structurally stable plastoglobules (Fig. 5f). Quantitative analysis confirmed an approximately 2.7-fold increase in chromoplast number and a 3.1-fold increase in plastoglobule number in OE1 relative to CK, with similar trends in OE2 (Fig. 5g and h). These results provide direct ultrastructural evidence that xanthophyll ester accumulation occurs within plastoglobules and contributes to their structural stability. Thus, the co-expression of *LbBCH1* and *LbXES2* promotes chromoplast and plastoglobule proliferation, stabilizes carotenoid ester sequestration, and ultimately enhances carotenoid accumulation in tomato fruit.

### LbBCH1 and LbXES2 overexpression impacts fruit flavor traits

Fruit flavor reflects an integrated perception of taste and aroma. Taste is largely determined by the relative proportions and absolute concentrations of soluble sugars, organic acids, and amino acids, whereas aroma is mainly contributed by volatile metabolites such as esters, alcohols, and terpenoids [29].

To further evaluate flavor-related traits in the transgenic fruits, we quantified major soluble sugars and organic acids at the ripe stage. Compared with the wild type (CK), fruits co-expressing *LbBCH1* and *LbXES2* (OE1 and OE2) showed altered levels of glucose, fructose, and sucrose, together with a change in citric acid content, resulting in a modified sugar-acid ratio (Fig. 6a). Glucose content was significantly lower in OE2 than in CK, while OE1 displayed an intermediate level (*P* <0.05). Fructose levels remained comparable across all lines. Notably, sucrose content was drastically and significantly elevated in OE1 (*P* <0.05), reaching approximately 3.0-fold higher than OE2 and CK, indicating a specific enhancement of sucrose accumulation. Consistent with this pattern, total soluble sugar content was significantly increased in transgenic fruits, with OE1 exhibiting the highest level, followed by OE2 and CK. In contrast, citric acid content was significantly reduced in both transgenic lines, with OE1 showing a highly significant reduction relative to CK. As a core flavor indicator, the sugar-acid ratio (total soluble sugar/citric acid) was dramatically improved in transgenic lines. OE1 achieved a ratio about 1.7-fold and 2.6-fold higher than OE2 and CK, respectively. These results demonstrate that co-overexpression of *LbBCH1* and *LbXES2* modulates sugar-acid metabolism, optimizes sugar–acid balance, and effectively improves tomato fruit flavor.

**Figure 6:**
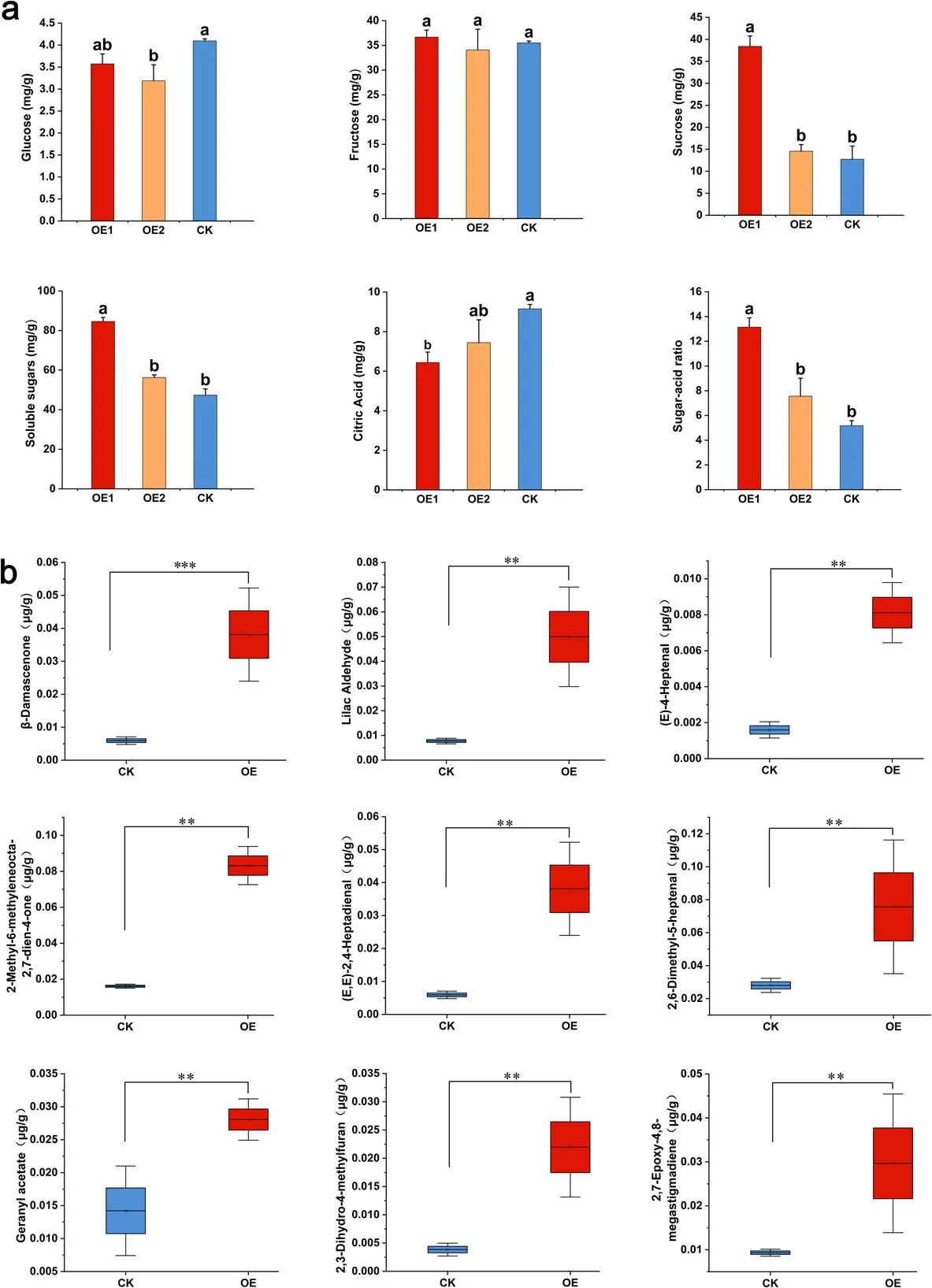
Evaluation of fruit flavor traits in transgenic tomatoes. (a) Analysis of sugar and citric acid contents and sugar–acid ratio in control (CK) and transgenic (OE1, OE2) tomato fruits. Sugar–acid ratio calculated as the ratio of total soluble sugar content to citric acid content. Data are presented as means ± SD. Different letters above the bars indicate significant differences at *P* <0.05 according to Tukey’s multiple range test. (b) Volatile apocarotenoids derived from carotenoids in ripe fruits of the control (CK) and transgenic lines (OE1). Asterisks indicate significant differences (^**^P <0.05; ^***^P <0.001). All T_1_ generation transgenic plants and non-transgenic negative control (CK) fruits were harvested at the fully ripe stage (breaker +20 d).

Volatile compounds in fruits are derived from multiple metabolic routes, including lipids, amino acids, terpenoids, and carotenoids. To determine whether carotenoid enrichment enhances the accumulation of volatile apocarotenoids, we analyzed the volatile profiles of transgenic and control fruits using GC–MS, with the representative transgenic line OE1 selected for subsequent experiments. As summarized in Supplementary Table S5, nine major classes of volatile compounds (classified as esters, alcohols, terpenoids, and aldehydes) differed significantly between transgenic and control fruits. In total, 191 compounds were increased (1.2- to 95.6-fold), while 120 compounds were reduced to 0%–50% of control levels in the transgenic lines (Table S5). We further examined nine representative carotenoid oxidative cleavage products. Among these, β-damascenone (derived from oxidative degradation of β-carotene/violaxanthin) and 2,3-dihydro-4-methylfuran (derived from oxidative degradation of lycopene) increased by 10.4- and 5.8-fold, respectively, relative to control. In addition, several other carotenoid-derived volatiles, including 2-methyl-6-methyleneocta-2,7-dien-4-one, geranyl acetate, 2,6-dimethyl-5-heptenal, lilac aldehyde, (E, E)-2,4-heptadienal, and 2,7-epoxy-4,8-megastigmadiene, were elevated by 2.3- to 5.5-fold compared with the control (Fig. 6b; Supplementary Table S6). These data indicate that engineering carotenoid accumulation and esterification can reshape downstream volatile metabolism, potentially contributing to altered aroma profiles in transgenic fruits.

## Discussion

In *L. barbarum* fruits, carotenoids primarily accumulate in esterified forms, with ZD being the most prominent [8]. As the dominant carotenoid ester, ZD is a major determinant of the characteristic coloration of ripe fruits and constitutes one of the most abundant nutritional components in *L. barbarum* [30]. Our ripening profiling analysis revealed a metabolic shift from free xanthophylls (e.g. zeaxanthin and lutein) toward their esterified derivatives starting at the turning stage. This pattern suggests that xanthophyll esterification is a ripening-associated process that facilitates carotenoid stabilization and storage. In this study, we identified LbXES2 as a xanthophyll esterification enzyme strongly associated with zeaxanthin esterification. We further provide biochemical and functional evidence supporting its role in both native and heterologous plant systems.

### LbXES2 is a plastid-localized xanthophyll acyltransferase with broad acceptor usage and a palmitoyl preference

Our functional assays demonstrate that LbXES2 can esterify multiple xanthophyll substrates and displays a strong preference for palmitoyl (C16:0) as the acyl donor, with myristoyl (C14:0) used less efficiently. Like other members of the XES-related acyltransferase family (Fig. S4), LbXES2 contains two distinguishable functional domains: a Hydrolase-4 domain and an acyltransferase domain related to DGAT. In Arabidopsis, PES1 and PES2 were identified as phytyl ester synthases that utilize medium-chain acyl donors (C10:0–14:0) [16]. In addition, tomato PYP1 as well as the rapeseed BjA02.PC1 and BjB04.PC2 have been shown to catalyze xanthophyll esterification in floral tissues, demonstrating a preference for diverse acyl donors [17, 22].

Our data identify LbXES2 as a plastid-localized xanthophyll acyltransferase involved in ZD biosynthesis in *L. barbarum*. Transient expression in *L. barbarum* and *N. benthamiana* leaves showed that LbXES2 has broad xanthophyll acceptor specificity and a strong preference for palmitoylation in planta. This catalytic profile was reproduced across multiple homologous and heterologous systems, providing convergent *in vivo* evidence for its biochemical properties. In these assays, not only lutein and zeaxanthin, but also violaxanthin and antheraxanthin were efficiently converted into their corresponding palmitate esters. Among these products, lutein palmitate accumulated to the highest level, suggesting that lutein may represent a favorable endogenous substrate for LbXES2. Notably, palmitate esters were the dominant products, while other acyl forms such as dimyristate esters remained at low levels, confirming the enzyme’s clear preference for palmitoyl donors. The enhanced ZD accumulation in the *N. benthamiana zep* background provided additional evidence that LbXES2 functions in plastids and directly utilizes xanthophyll substrates within their native subcellular compartment.

Although *in vitro* enzymatic assays were not successfully established, mainly because of protein solubility and substrate-related limitations in prokaryotic expression systems, the consistent *in vivo* results from multiple assay platforms strongly support the proposed activity of LbXES2. Future studies with optimized heterologous expression and enzyme assay systems are needed to define its catalytic mechanism and substrate preferences in greater biochemical detail.

### ZD accumulation is strongly substrate-dependent, and LbXES2 preferentially favors diesterification when zeaxanthin supply is sufficient

Our results consistently indicate that ZD accumulation is strongly constrained by zeaxanthin availability and that LbXES2 preferentially catalyzes diesterification when sufficient substrate is present. In *L. barbarum* leaves, ZD was undetectable in control plants. Overexpression of *LbXES2* alone produced only trace amounts of zeaxanthin monopalmitate, whereas co-expression with *LbBCH1* markedly induced ZD accumulation. Together, these results suggest that zeaxanthin supply is a major limiting factor for LbXES2-mediated ZD biosynthesis. By promoting the conversion of β-carotene to zeaxanthin, LbBCH1 expands the substrate pool available to LbXES2 and thereby enables efficient ZD formation. This finding highlights the importance of precursor availability in determining the metabolic output of LbXES2. Evidence from *N. benthamiana* further corroborates this substrate-dependent model. Expression of *LbXES2* in *zep* mutants of *N. benthamiana*, which accumulate elevated levels of zeaxanthin due to impaired epoxidation, led to substantially higher ZD production than in *L. barbarum* leaves or wild-type *N. benthamiana*. Moreover, co-expression of *LbBCH1* and *LbXES2* in *N. benthamiana* further increased ZD accumulation. Together, these results demonstrate a close dependence of ZD formation on zeaxanthin supply.

Notably, the preferential accumulation of ZD over zeaxanthin monopalmitate in *zep* mutant leaves directly demonstrates the intrinsic catalytic preference of LbXES2 for diesterification under sufficient substrate. This preference redirects metabolic flux toward ZD rather than its monoester precursor, highlighting a key biochemical characteristic of LbXES2 that becomes evident only under conditions of high zeaxanthin availability. The *de novo* biosynthesis of ZD in tomato fruits through stable co-overexpression of *LbBCH1* and *LbXES2* further validates this framework in a heterologous fleshy fruit system and confirms that enhancing zeaxanthin enables LbXES2 to efficiently channel metabolic flux toward ZD. Collectively, these findings support a model in which ZD accumulation is governed jointly by precursor availability and xanthophyll esterification capacity, which provides a mechanistic basis for engineering high-value carotenoid esters in horticultural crops.

### Engineering the LbBCH1–LbXES2 module in tomato links carotenoid enrichment to improved fruit flavor traits

Fruit flavor traits are primary drivers of global fruit consumption, yet inferior flavor in modern commercial tomato cultivars remains a major source of consumer dissatisfaction. Volatile organic compounds (VOCs) are critical contributors to tomato flavor and consumer acceptability and can enhance perceived sweetness [31]. In particular, apocarotenoid volatiles impart characteristic fruity and floral aroma notes and positively influence consumer preference [31, 32]. Many volatiles arise from interconnected metabolic networks, and historical breeding has inadvertently introduced alleles that compromise quality. For example, a mutation in the Golden 2-like transcription factor alters chlorophyll accumulation as well as distribution, resulting in reduced sugars and carotenoids in ripe fruits [33]. Conversely, selection for the deep red trait associated with high lycopene has been linked to increased accumulation of 6-methyl-5-hepten-2-one, a lycopene-derived aroma compound [34]. Vogel et al. [35] further demonstrated that carotenoid perturbations in mutants can impair apocarotenoid volatile formation and alter sensory attributes.

Notably, co-expression of *LbBCH1* and *LbXES2* not only facilitated the accumulation of esterified xanthophylls, including ZD, but also elevated the overall pool of free carotenoids. This expansion of the carotenoid reservoir likely provides a larger substrate supply for apocarotenoid volatile formation (Fig. 6b). Consistent with this possibility, volatile profiling revealed elevated levels of β-damascenone, 2,3-dihydro-4-methylfuran, 2-methyl-6-methyleneocta-2,7-dien-4-one, and 2,7-epoxy-4,8-megastigmadiene. These compounds are apocarotenoid volatiles derived from the degradation of β-carotene, lycopene, and violaxanthin, and they contribute characteristic rose-like, fruity, and fatty aromas in tomato fruit [34].

Carotenoid-derived VOCs can be generated enzymatically by carotenoid cleavage dioxygenases (CCDs) or produced nonenzymatically through oxidative cleavage of linear and cyclic carotenoids [35, 36]. In tomato, fruit-expressed CCDs contribute substantially to isoprenoid volatiles formation. Ilg et al. [37] showed that SlCCD1A and SlCCD1B display broad substrate specificity *in vitro* and generate the majority of tomato isoprenoid volatiles, while SlCCD7 cleaves cyclic and acyclic carotenoids and is strongly expressed in fruits [38]. By contrast, relatively low expression of LbCCD1, LbCCD4, and LbCCD7 has been reported in *L. barbarum* fruits [27], which may help explain why wolfberry can accumulate abundant carotenoids yet exhibit comparatively weak aroma. This comparison emphasizes that fruit flavor is not determined solely by carotenoid abundance but also by the expression and activity of downstream cleavage pathways. Therefore, co-overexpression of *LbXES2* and *LbBCH1* to boost carotenoid biosynthesis and accumulation represents a promising genetic strategy for targeted optimization of tomato flavor profiles by simultaneously increasing carotenoid pools and supporting downstream apocarotenoid volatile formation.

## Conclusion

Overall, this study represents the first systematic isolation and characterization of *LbBCH1* and *LbXES2* from *L. barbarum*, a typical ZD-accumulating plant of significant medicinal and nutritional value. Our data identified LbXES2 as a xanthophyll acyltransferase associated with ZD biosynthesis and support a role for LbBCH1-mediated zeaxanthin supply in promoting ester accumulation. Heterologous reconstruction of the LbBCH1–LbXES2 pathway in tomato enabled *de novo* ZD production, altered flavor-related metabolite profiles, and increased plastoglobule abundance, suggesting enhanced carotenoid storage capacity. Although direct loss-of-function studies in *L. barbarum* would further strengthen causal inference, such experiments are currently constrained by the limited efficiency of stable transformation and the long juvenile growth phase of this species. Nevertheless, the combined developmental, cellular, biochemical, transient and heterologous expression evidence provides strong functional support for the roles of LbXES2 and LbBCH1 in ZD-associated metabolism. These findings offer useful molecular tools and a transferable strategy for engineering high-value esterified carotenoids in horticultural crops.

## Materials and methods

### Plant growth and sampling

The plant materials used in this study were obtained from *L. barbarum* Ningqi 1 (N1), cultivated in the experimental farm of Ningxia University. Fruits of N1 were harvested at four distinct developmental stages: 12 days after flowering (S1, green stage), 19 days after flowering (S2, turning stage), 26 days after flowering (S3, red stage), and 33 days after flowering (S4, ripe stage). All harvested fruits and leaves were immediately flash-frozen in liquid nitrogen and stored at −80°C for subsequent analysis.

For transient expression assays, we used four-week-old N1 and tobacco (*N. benthamiana*) plants cultivated in a controlled growth chamber under conditions of 16 h light/8 h dark, 25°C ± 2°C, and 40%–60% humidity.

### *LbXES2* gene cloning and sequence analysis

Homologous sequences of LbXES were identified through BLAST search against the *L. barbarum* genome database (BIOproject/Lycium barbarum isolate: ZL-2021 (goji berry); total nucleotides: 69 899), using *Arabidopsis thaliana* At1g54570 and *Solanum lycopersicum* SIPYP1 Solyc01g098110.2 as query sequences.

Gene-specific primers were designed to amplify the full-length coding sequences of candidate genes (Supplementary Table S7), and polymerase chain reaction (PCR) amplification was conducted using high-fidelity ApexHF HS DNA Polymerase FS (Accurate Biotechnology, China). The resulting amplicons were verified by Sanger sequencing (Biosune Biotechnology, China), and sequence analysis was performed using DNASTAR Lasergene SeqMan software.

For comparative analysis, additional XES sequences from various plant species were retrieved from the National Center for Biotechnology Information database (https://www.ncbi.nlm.nih.gov/) and used for multiple sequence alignment. A maximum-likelihood phylogenetic tree was constructed using Molecular Evolutionary Genetics Analysis software, incorporating bootstrap analysis based on 1000 replicates to assess node support [39]. The evolutionary relationships between LbXES and XES proteins from distantly related plant species were analyzed by examining distinct clades within the phylogenetic tree.

### LbBCH1 and LbXES2 proteins subcellular localization analysis

The full-length open reading frames of *LbXES* were cloned into the pEAQ-SalI transient expression gateway vector, incorporating a C-terminal GFP fusion tag. The construct was transformed into *Agrobacterium tumefaciens* GV3101 for transient expression in *N. benthamiana* leaves. Fluorescence in *N. benthamiana* leaves was visualized using a Leica STELLARIS 5 confocal laser scanning microscope and analyzed with Leica LAS-X software. The GFP fluorescence was excited using a 488 nm laser, with emission signals collected between 500 and 560 nm. Chloroplast autofluorescence was excited using a 590-nm laser, and emitted signals were collected across a 610–750 nm spectral window. Images presented in all figures are representative of results obtained from at least five independent experimental replicates.

### *Escherichia coli-*based pigment complementation assay

The CDS of LbBCH1 and LbXES2 without their predicted signal peptides were cloned into the pET-32a vector. The recombinant plasmid pET-LbBCH1 was co-transformed into *E. coli* BL21(DE3) with pAC-BETA, a plasmid carrying the carotenoid biosynthetic genes *crtE*, *crtB*, *crtI*, and *crtY* from *Pantoea agglomerans* for β-carotene production. Similarly, pET-LbXES2 was co-transformed into *E. coli* BL21(DE3) with pAC-ZEAX, which harbors *crtE*, *crtB*, *crtI*, *crtY*, and *crtZ* to enable zeaxanthin accumulation. Cell growth, protein expression, and pigment analysis were performed as described [25, 26] with minor modifications.

### *LbBCH1* and *LbXES2* transient expression in *N. benthamiana* and *L. barbarum*

The target genes *LbBCH1* and *LbXES2* were cloned into the pEAQ-XhoI expression vector, with the mNeonGreen fluorescent protein gene serving as a negative control due to its monomeric yellow-green fluorescence characteristics. The individual constructs were separately transformed into *Agrobacterium tumefaciens* GV3101, and positive clones were selected on Luria-Bertani (LB) plates supplemented with 50 μg/ml kanamycin and 20 μg/ml rifampicin, followed by incubation at 28°C for 2 days. The primer sequences used for these constructs are listed in Supplementary Table S7. Single recombinant colonies were inoculated into LB liquid medium supplemented with the corresponding antibiotics (50 μg/ml kanamycin and 20 μg/ml rifampicin) and cultured overnight at 28°C with vigorous shaking at 220 rpm. The Agrobacterium tumefaciens cells were collected by centrifugation at 6000 rpm for 10 min at room temperature, after which the spent medium was carefully aspirated. The bacterial pellet was subsequently resuspended in 5 ml of filter-sterilized induction medium (10 mM MES, pH 5.6, 10 mM MgCl_2_, and 0.20 mM acetosyringone). *Agrobacterium tumefaciens* suspensions (OD_600_ = 0.2 for each strain) were infiltrated into the abaxial leaf surfaces using a needleless syringe. Leaf samples (*n* = 6 biological replicates) were harvested at 6 days postinfiltration (dpi).

### *LbBCH1* and *LbXES2* heterologous expression in tomato

The CDS of *LbBCH1* (without stop codon) and *LbXES2* (full-length) were cloned into the binary vector pNULPGE200, flanked by the fruit-specific E8 promoter and the Arabidopsis HSP18.2 terminator. To enable coordinated expression, the genes were linked via a 2A self-cleaving peptide sequence (Fig. S5). We selected a yellow-fruited variety of *Solanum lycopersicum* var*. cerasiforme* ‘Yellow Fruit Cherry Tomato’, which contains minimal levels of esterified carotenoids (Table S4), for genetic transformation. Agrobacterium-mediated transformation and subsequent transgenic plant characterization were performed according to established methods [40]. Putative transgenic plants were selected under 50 μg/ml kanamycin pressure. Surviving plantlets were subjected to genomic PCR analysis using transgene-specific primers (Supplementary Table S7) to confirm successful integration of both target genes. Genomic DNA was isolated from the regenerated plantlets and WT controls using the EasyPure Plant Genomic DNA Kit (TransGen Biotech, China).

### RNA extraction and quantitative real-time PCR analysis

Fruits from all T_1_ generation transgenic plants and the nontransgenic negative control (CK) were harvested at the fully ripe stage (breaker + 20 d) for RNA isolation using the RNAprep Pure Plant Kit (Tiangen, China). RNA concentration and purity were quantified using a NanoDrop ND-2000 spectrophotometer (Thermo Fisher Scientific, USA). All RNA samples were normalized to a concentration of 60 ng/μl and reverse-transcribed into first-strand cDNA using the HiScript II first Strand cDNA Synthesis Kit (with gDNA wiper) (Vazyme, China), following the manufacturer’s protocol. Relative transcript abundance was quantified by qRT-PCR using ChamQ Universal SYBR qPCR Master Mix (Vazyme, China) on a qTOWER3 thermal cycler (Analytik Jena, Germany). Gene-specific primers (Supplementary Table S7) were used for amplification, with three technical replicates performed for each sample to ensure reproducibility.

### TEM analysis

Fruits from T₁ transgenic lines and nontransgenic control (CK) plants were harvested at two late ripening stages: 20 days after breaker stage (Br + 20 d, Stage 1) and 25 days after breaker stage (Br + 25 d, Stage 2). Fruits sampled from the these two developmental stages of CK and T₁ transgenic plants were subsequently subjected to TEM analysis, which was performed according to Li et al. [41].

### Quantification of soluble sugars and citric acid

Fruits from all T_1_ transgenic plants and a nontransgenic negative control (CK) were harvested at the fully ripe stage (breaker + 20 d). Contents of sucrose, glucose, fructose, and total soluble sugars were determined using commercial kits BC2465, BC2505, BC2455, and BC0030 (Solarbio, Beijing, China), respectively. Citric acid content was quantified using kit BC2155 (Solarbio, Beijing, China). All operations followed the manufacturer’s protocols, and three biological replicates were performed for each sample.

### Carotenoid profiling analysis

The samples were freeze-dried and homogenized by grinding at 30 Hz for 1.5 min, 50 mg of the powdered sample was accurately weighed and mixed with 0.5 ml of a ternary solvent system consisting of n-hexane/acetone/ethanol (1:1:1, v/v/v). The mixture was vortexed vigorously for 20 min at room temperature, followed by centrifugation at 12 000 rpm for 5 min at 4°C. The supernatants were evaporated to dryness under a gentle stream of nitrogen and reconstituted in 100 μl dichloromethane. The solution was filtered through a 0.22 μm organic membrane prior to analysis. The sample extracts were analyzed using an ultra-high performance liquid chromatography-atmospheric pressure chemical ionization-tandem mass spectrometry (UPLC-APCI-MS/MS) system (ExionLC™ AD UPLC coupled to an Applied Biosystems 6500 Triple Quadrupole mass spectrometry, SCIEX). Separation was achieved using a YMC C30 analytical column (3 μm particle size, 100 mm × 2.0 mm i.d.) maintained at 28°C. Mobile phase A: methanol:acetonitrile (1:3, v/v) containing 0.01% BHT and 0.1% formic acid, while mobile phase B: methyl tert-butyl ether with 0.01% BHT. A gradient elution was applied: 0–3 min, 0% B (isocratic), 3–5 min, 0%–70% B (linear gradient); 5–9 min, 70%–95% B (linear gradient), 9–10 min: 95%–0% B (rapid equilibration); 10–11 min, 0% B (column re-equilibration). The flow rate was maintained at 0.8 ml/min with an injection volume of 2 μl. The APCI source was operated in positive ionization mode (APCI+) with the following optimized parameters: ion source temperature, 350°C; curtain gas, 25.0 psi (nitrogen). Carotenoid analysis was performed using scheduled multiple reaction monitoring to enhance detection sensitivity and selectivity. All analyzes were conducted in triplicate to ensure methodological reproducibility. Metabolite identification was performed by matching retention times (±0.2 min), accurate mass measurements (≤5 ppm), and MS/MS fragmentation patterns with reference standards from the MetWare database (MetWare Biotechnology, China).

### Analysis of volatile compounds

Fruits from all T_1_ transgenic plants and a nontransgenic negative control (CK) were harvested at the fully ripe stage (breaker + 20 d), weighed, immediately frozen in liquid nitrogen, and stored at −80°C until use. Samples were ground to a powder in liquid nitrogen. Five hundred milligrams of the sample was transferred immediately to a 20 ml headspace vial (Agilent, Palo Alto, CA, USA) containing 2 ml NaCl-saturated solution to inhibit any enzyme reactions. The vials were sealed using crimp-top caps with polytetrafluoroethylene (TFE)-silicone headspace septa (Agilent). At the time of solid-phase microextraction (SPME) analysis, each vial was placed at 60°C for 5 min, then a SPME Arrow (Agilent) of 120 μm DVB/CWR/PDMS was exposed to the headspace of the sample for 15 min at 60°C. After sampling, desorption of the VOCs from the SPME Arrow coating was carried out in the injection port of the GC apparatus (Model 8890; Agilent) at 250°C for 5 min. The identification and quantification of VOCs were carried out using an Agilent Model 8890 GC and a 7000D mass spectrometer (Agilent), equipped with a 30 m × 0.25 mm × 0.25 μm DB-5MS (5% phenyl-polymethylsiloxane) capillary column. Helium was used as the carrier gas at a linear velocity of 1.2 ml/min. The injector temperature was kept at 250°C. The oven temperature was programmed from 40°C (3.5 min), increasing at 10°C/min to 100°C, at 7°C/min to 180°C, and at 25°C/min to 280°C, with a 5 min hold. Mass spectra were recorded in electron impact (EI) ionization mode at 70 eV. The quadrupole mass detector, ion source, and transfer line temperatures were set at 150, 230, and 280°C, respectively. The MS was operated in selected ion monitoring mode for the identification and quantification of analytes.

### Statistical analysis

All experiments were performed in three independent replicates. Statistical analyzes and visualization were performed using Origin 9.1 (OriginLab Corp., Northampton, MA, USA). Results are presented as mean values ± standard deviation (SD). One-way analysis of variance (ANOVA) followed by *post hoc* Tukey’s test was employed to determine statistical significance (*P* < .05). In graphical representations, different lowercase letters (a, b, c, etc.) denote statistically significant differences between groups.

## Supplementary Material

Web_Material_uhag228

## Data Availability

All the data supporting the findings of this study are found within the manuscript and its supplementary data, and are available from the corresponding author upon request. The data used for generating graphs are shown in the source data for figures, supplementary figures, and tables.xlsx.
