# Supplementary material for: Engineering xanthophyll esterification: LbXES2 mediates zeaxanthin dipalmitate biosynthesis and remodels fruit flavor metabolism
Source: Hortic Res. 2026 Jun 10;13(11):uhag228. doi: 10.1093/hr/uhag228 (PMC13722871; doi:10.1093/hr/uhag228)
Supplement: Supplementary file 1 [file web_material_uhag228.zip › Suplemental figures .docx]

**Figure S1** Simplified and modified representation of the known carotenoid biosynthetic pathway leading to zeaxanthin dipalmitate. Dashed lines indicate possible alternative route(s). GGPPS, geranylgeranyl diphosphate synthase; PSY, phytoene synthase; PDS, phytoene desaturase; Z-ISO, ζ-carotene isomerase; ZDS, ζ-carotene desaturase; CRTISO, carotene isomerase; LCYe, lycopene ε-cyclase; LCYb, lycopene β-cyclase; CYP97, cytochrome P450-type monooxygenase 97; BCH, β-carotene hydroxylase; XES, xanthophyll esterases; ZEP, zeaxanthin epoxidase; VDE, violaxanthin de-epoxidase

**Figure S2** Xanthophyll contents and their changes in *Lycium barbarum* leaves and fruits across different fruit development stages.

(a) Xanthophyll contents in leaves.

(b) Changes in zeaxanthin and lutein contents in leaves and during different fruit development stages.

Values are presented as mean ± SE (n = 3). Statistical significance was evaluated by one-way analysis of variance (ANOVA) followed by Duncan's multiple range test. Different letters above the error bars indicate significant differences at *p* < 0.05.

**Figure S3** Phylogenetic analysis of the BCH and XES.

Unrooted maximum-likelihood phylogenetic trees of BCH (a) and XES (b) were reconstructed from homologous sequences across diverse taxa using MEGA 11.0. Branch support values (shown at nodes) represent bootstrap percentages from 1,000 replicates.

**Figure S4** Multiple sequence alignments of LbBCH and selected homologous proteins. *Arabidopsis thaliana*, NP_001320065.1; *Arabidopsis thaliana*, NP_001119420.1; *Malus domestica*, XP_008343769.2; *Prunus dulcis*, BBG98323.1; *Citrus sinensis*, NP_001275830.1 / CsBCH1; *Ricinus communis*, XP_002513654.1; *Zea mays*, NP_001105907.1; *Zea mays*, NP_001105865.2; *Daucus carota*, UWK22228.1 / DcBCH1; *Apium graveolens*, QDC33551.1; *Actinidia chinensis var. chinensis*, PSS04390.1 / AcBCH2; *Actinidia chinensis var. chinensis*, PSS33035.1 / AcBCH1; *Gentiana lutea*, BAE92729.1; *Lycium barbarum*, XP_060203720.1 / LbBCH1; *Solanum lycopersicum*, NP_001265981.1 / SlBCH1; *Petunia x hybrida*, QBC36226.1; *Lycium barbarum*, XP_060211839.1 / LbBCH; *Solanum lycopersicum*, NP_001234348.1 / SlBCH2.

**Figure S5** Multiple sequence alignment of LbXES2 and selected homologous proteins. Brassica rapa, XP_ 009129473.1; *Brassica napus*, XP_ 013698349.2; *Brassica juncea*, BjuB027334/BjPC2; *Arabidopsis thaliana*, AT3G26820; *Arabidopsis thaliana*, AT3G26820; *Citrus x clementina*, ESR60233.1; *Solanum lycopersicum*, XP_ 004231751.1; *Arabidopsis thaliana*, At1g54570 / AtPES1; *Tagetes erecta*, BBB44441.1/TeXES; *Sesamum indicum*, XP_011081015.1; *Ipomoea obscura*, BBB44442.1 / IoXES; *Calibrachoa hybrid cultivar*, BBB44444.; *Petunia x hybrida*, BBB44443.1; *Lycium barbarum*, XP_060218063.1 / LbXES1; *Lycium barbarum*, XP_060218059.1 / LbXES2; *Capsicum baccatum*, PHT41226.1; *Solanum lycopersicum*, XP 004230141.1 / *SlPYP1; Solanum tuberosum*, XP_006347827.1.

**Figure S6** Schematic diagram of the E8- *LbXES2/LbBCH1* binary vector and PCR screening of screening transgenic tomato plants

(a) Schematic diagram of E8- *LbXES2/LbBCH1* binary vector used for *Agrobacterium*-mediated transformation in tomato. Arrows indicate the E8 promoter; *LbXES2* and *LbBCH1* are linked by P2A peptide; TAtHSP18.2, *AtHSP18.2* terminator.
(b) Agarose gel electrophoresis analysis of PCR products for transgenic tomato plants. M, DNA marker; P, plasmid positive control; N, non-transgenic negative control; lanes 1-5, independent transgenic plants (1-5)
