# Supplementary material for: Engineering xanthophyll esterification: LbXES2 mediates zeaxanthin dipalmitate biosynthesis and remodels fruit flavor metabolism
Source: Hortic Res. 2026 Jun 10;13(11):uhag228. doi: 10.1093/hr/uhag228 (PMC13722871; doi:10.1093/hr/uhag228)
Supplement: Supplementary file 1 [file web_material_uhag228.zip › Suplemental tables.docx]

**Table S1** Carotenoid contents in transient expression assays of *LbXES2* and *LbXES2/ LbBCH1* in *L. barbarum* leaves (Unit: μg/g dry weight, DW)

| Compounds | CK | LbXES2 | LbXES2/LbBCH1 |
| --- | --- | --- | --- |
| zeaxanthin | 13.38 ± 2.6 | 54.59 ± 8.22 | 3.74 ± 0.24 |
| violaxanthin | 172.06 ± 34.28 | 66.69 ± 39.71 | 148.75 ±30.01 |
| β-cryptoxanthin | 11.90 ± 3.16 | 16.43 ± 3.80 | 6.45 ± 0.14 |
| antheraxanthin | 21.62 ± 2.8 | 15.32 ± 3.24 | 8.86 ± 1.71 |
| β-carotene | 290.15 ± 32.95 | 206.99 ± 12.13 | 260.44 ± 24.15 |
| lutein dimyristate | 0.08 ± 0.00 | 1.86 ± 0.06 | 0.16 ± 0.07 |
| lutein palmitate | 0.69 ± 0.06 | 16.60 ± 0.73 | 4.87 ± 0.16 |
| lutein dipalmitate | 0.11 ± 0.01 | 1.50 ± 0.05 | N/A |
| zeaxanthin palmitate | N/A | 0.16 ± 0.02 | N/A |
| antheraxanthin dipalmitate | N/A | 2.21 ± 0.14 | N/A |
| violaxanthin dipalmitate | N/A | 4.61 ± 0.23 | 1.09 ± 0.28 |
| zeaxanthin dipalmitate | N/A | N/A | 0.48 ± 0.03 |

Data are presented as mean ± SE (n = 3 biological replicates). The ‘CK’ represents the empty vector (CK) control, ‘LbXES2’ represents the expression group of *LbXES2* alone, and ‘LbXES2/LbBCH1’ represents the co-expression group of *LbXES2* and *LbBCH1*.

**Table S2** Carotenoid contents in transient expression assays of *LbXES2* and *LbXES2/ LbBCH1* in wild-type *N. tabacum* leaves (Unit: μg/g dry weight, DW)

| Compounds | CK | LbXES2 | LbXES2/LbBCH1 |
| --- | --- | --- | --- |
| zeaxanthin | 5.82 ± 0.62 | 22.77 ± 0.47 | 70.23 ± 0.68 |
| violaxanthin | 178.94 ± 11.45 | 340.03 ± 12.09 | 540.04 ±14.18 |
| β-cryptoxanthin | 9.44 ± 0.33 | 8.12 ± 0.15 | 17.21 ± 0.95 |
| antheraxanthin | 10.82 ± 1.49 | 61.88 ± 2.66 | 241.27 ± 3.25 |
| β-carotene | 567.49 ± 7.42 | 483.63 ± 4.77 | 130.95 ± 4.33 |
| lutein dimyristate | 0.03 ± 0.00 | 1.21 ± 0.14 | 0.25 ± 0.05 |
| lutein palmitate | 0.25±0.03 | 13.22 ± 0.94 | 6.69 ± 0.24 |
| zeaxanthin palmitate | 0.14±0.02 | 0.42 ± 0.04 | 0.35 ± 0.03 |
| violaxanthin palmitate | N/A | 2.88 ± 0.14 | 7.74 ± 0.46 |
| antheraxanthin dipalmitate | N/A | 1.96 ± 0.15 | 1.19 ± 0.10 |
| violaxanthin dipalmitate | N/A | 3.85 ± 0.22 | 3.23 ± 0.23 |
| zeaxanthin dipalmitate | N/A | 0.30 ± 0.01 | 0.48 ± 0.04 |

Data are presented as mean ± SE (n = 3 biological replicates). The ‘CK’ represents the empty vector (CK) control, ‘LbXES2’ represents the expression group of *LbXES2* alone, and ‘LbXES2/LbBCH1’ represents the co-expression group of *LbXES2* and *LbBCH1*.

**Table S3** Carotenoid contents in transient expression assays of *LbXES2* and *LbXES2/ LbBCH1* in *N. benthamiana* *zep* knockout mutant leaves (Unit: μg/g dry weight, DW)

| Compounds | CK | LbXES2 | LbXES2/LbBCH1 |
| --- | --- | --- | --- |
| zeaxanthin | 632.54 ± 63.78 | 575.45 ± 15.61 | 779.51 ± 10.65 |
| violaxanthin | N/A | N/A | N/A |
| β-cryptoxanthin | 8.05 ± 0.52 | 3.94 ± 0.23 | 7.79 ± 0.42 |
| antheraxanthin | N/A | N/A | N/A |
| β-carotene | 225.72 ± 12.31 | 140.29 ± 0.26 | 129.15 ± 12.05 |
| lutein dimyristate | N/A | N/A | N/A |
| lutein palmitate | 0.55 ± 0.01 | 3.08 ± 0.05 | 1.58 ± 0.10 |
| zeaxanthin dimyristate | N/A | 0.40 ± 0.02 | 0.16 ± 0.01 |
| β-cryptoxanthin palmitate | N/A | 0.35 ± 0.00 | 0.34 ± 0.02 |
| lutein dipalmitate | N/A | N/A | N/A |
| zeaxanthin palmitate | 0.63 ± 0.37 | 0.77 ± 0.06 | 1.09 ± 0.02 |
| antheraxanthin dipalmitate | N/A | N/A | N/A |
| violaxanthin dipalmitate | N/A | N/A | N/A |
| zeaxanthin dipalmitate | 0.00 | 1.13 ± 0.05 | 1.54 ± 0.03 |

Data are presented as mean ± SE (n = 3 biological replicates). The ‘CK’ represents the empty vector (CK) control, ‘LbXES2’ represents the expression group of *LbXES2* alone, and ‘LbXES2/LbBCH1’ represents the co-expression group of *LbXES2* and *LbBCH1*.

**Table S4** Carotenoid profile and contents in yellow cherry tomatoes (Unit: μg/g dry weight, DW)

| **Compounds** | **lycopene** | **β-carotene** | **zeaxanthin** | **lutein** | **violaxanthin** | **lutein**  **palmitate** | **lutein**  **dipalmitate** | **zeaxanthin**  **palmitate** | **zeaxanthin**  **dipalmitate** | **violaxanthin**  **palmitate** | **violaxanthin**  **dipalmitate** |
| --- | --- | --- | --- | --- | --- | --- | --- | --- | --- | --- | --- |
| **Content（ug/g)** | **0.09±0.03** | **2.44±0.2** | **3.32±0.34** | **25.35±5.44** | **0.46±0.11** | **0.17±0.05** | **0.04±0.00** | **0.00±0.00** | **0.00±0.00** | **0.25±0.05** | **0.00±0.00** |

Data are presented as mean ± SE (n = 3 biological replicates).

**Table S5** Volatile compounds tentatively identified in whole fruits.

| **Compounds** | **All significant difference** | **Up/** **Fold_Change** | **Down/** **Fold_Change** |
| --- | --- | --- | --- |
| Ester | 57 | 30, 3.5-22.1 | 27, 0.0-0.2 |
| Amine | 13 | 7, 1.5-2.4 | 6, 0.1-0.2 |
| Alcohol | 58 | 36, 3.3-20.6 | 22, 0.0-0.3 |
| Terpenoids | 57 | 34, 2.0-10.4 | 23, 0.0-0.4 |
| Aromatics | 5 | 4, 5.0-5.3 | 1, 0.2 |
| Phenol | 18 | 10, 1.2-2.7 | 8, 0.1-0.2 |
| Aldehyde | 40 | 27, 2.3-6.3 | 13, 0.0-0.1 |
| Acid | 18 | 9, 2.4-7.0 | 9, 0.2-0.5 |
| Ketone | 45 | 34, 2.3-95.6 | 11, 0.2-0.4 |

Columns 3 and 4: the value before the comma denotes the number of detected compounds, whereas that after the comma represents the fold change of increase or decrease in compound content.

**Table** **S6** VOC contents in tomato fruits co-overexpressing *LbXES2* and *LbBCH1* (Unit: μg/g fresh weight, FW)

| Compounds | CK | OE1 |
| --- | --- | --- |
| β-Damascenone | 0.049 ± 0.00 | 0.44 ± 0.025 |
| Lilac Aldehyde | 0.01 ± 0.00 | 0.05 ± 0.008 |
| (E)-4-Heptenal | 0.001 ± 0.00 | 0.008 ± 0.00 |
| 2-Methyl-6-methyleneocta-2,7-dien-4-one | 0.016 ± 0.00 | 0.083 ± 0.004 |
| (E,E)-2,4-Heptadienal | 0.006 ± 0.00 | 0.038 ± 0.00 |
| 2-Methyl-6-methyleneocta-2,7-dien-4-one | 0.016 ± 0.00 | 0.085 ± 0.004 |
| Geranyl acetate | 0.014 ± 0.002 | 0.028 ± 0.001 |
| 2,3-Dihydro-4-methylfuran | 0.004 ± 0.00 | 0.022 ± 0.003 |
| 2,7-Epoxy-4,8-megastigmadiene | 0.009 ± 0.00 | 0.03 ± 0.006 |

Data are presented as mean ± SE (n = 3 biological replicates).

**Table S7** Sequences of primers used in this study.

| Primer name | Primer sequence(5'-3') | Function |
| --- | --- | --- |
| LbBCH1CDS-F | ATGGCTGCCGGAGTTTCAG | Full lengths clone |
| LbBCH1CDS-R | TCATAATCCCTTAGAAATTTTAATTC | Full lengths clone |
| LbXES1CDS-F | ctgcccaaattcgcgctcgagATGGCTTCTCTTCTGCAAAATTTC | Full lengths clone for pEAQ |
| LbXES1CDS-R | tgaaaccagagttaactcgagCTAATAATCAAATGTTGGGACATCAT | Full lengths clone for pEAQ |
| LbXES2CDS-F | ctgcccaaattcgcgctcgagATGAACAGACACCAAACTAGTTCC | Full lengths clone for pEAQ |
| LbXES2CDS-R | tgaaaccagagttaactcgagTTAATAATCAAATGTTGGGACATC | Full lengths clone for pEAQ |
| LbBCH1F(Sub) | aaattcgcgctcgaggtcgacATGGCTGCCGGAGTTTCAG | Subcellular localization |
| LbBCH1R(Sub) | acctttgctgaccatgtcgacTAATCCCTTAGAAATTTTAATTC | Subcellular localization |
| LbBCH2F(Sub) | aaattcgcgctcgaggtcgacATGGCAGCCGCCAGAATT | Subcellular localization |
| LbBCH2R(Sub) | acctttgctgaccatgtcgacTGATCCCTTTGAAAGTTTTATCC | Subcellular localization |
| LbXES1F(Sub) | aaattcgcgctcgaggtcgacATGGCTTCTCTTCTGCAAAATTTC | Subcellular localization |
| LbXES1R(Sub) | acctttgctgaccatgtcgacATAATCAAATGTTGGGACATCATT | Subcellular localization |
| LbXES2F(Sub) | aaattcgcgctcgaggtcgacATGAACAGACACCAAACTAGTTCC | Subcellular localization |
| LbXES2R(Sub) | acctttgctgaccatgtcgacATAATCAAATGTTGGGACATCATC | Subcellular localization |
| LbBCH1qF | GCTACCTCCCGAACGTTTTG | qRT-PCR |
| LbBCH1qR | GCGGGTTTTGGGCCTAGA | qRT-PCR |
| LbBCH2qF | GCCCAAAACCCACCTCAGT | qRT-PCR |
| LbBCH2qR | GAATTGCGCCCAAATTAGGA | qRT-PCR |
| LbXES1qF | CGGATAAGGCGTGATAACGAA | qRT-PCR |
| LbXES1qR | GTCCGCCCTGACTGCTAAAC | qRT-PCR |
| LbXES2qF | ATATTATGGTTTCTCTTCCGCAAAA | qRT-PCR |
| LbXES2qR | GCCTGGGTGACGAGCAAA |  |
| 32b-BCHnospF | taagaaggagatatacatatgGGATGCTAGGAAAACTGAGATTG | Clone for pET-32b |
| 32b-BCHnospR | gtggtggtggtggtgctcgagTAATCCCTTAGAAATTTTAATTC | Clone for pET-32b |
| 32b-XES2nospF | taagaaggagatatacatatgAAGAGCAAGGAGGACATGCGAAACAAG | Clone for pET-32b |
| 32b-XES2nospR | gtggtggtggtggtgctcgagATAATCAAATGTTGGGACATCATC | Clone for pET-32b |
| E8F | CAACCTCCATGCCACTTGTCC | Overexpression plant identification |
| LbXES2R(244） | CAAGGTTGAACAAAGCGTACAGG | Overexpression plant identification |
